# Association of urinary sodium and potassium excretion with systolic blood pressure in the Dietary Approaches to Stop Hypertension Sodium Trial

**DOI:** 10.1038/s41371-020-0375-8

**Published:** 2020-07-13

**Authors:** Parul Chaudhary, Richard D. Wainford

**Affiliations:** 1 Department of Pharmacology and Experimental Therapeutics, Boston University School of Medicine, Boston, MA USA; 2 The Whitaker Cardiovascular Institute, Boston University, Boston, MA USA; 3 Department of Health Sciences, Boston University Sargent College, Boston, MA USA

**Keywords:** Risk factors, Hypertension

## Abstract

The 2019 National Academy of Science, Engineering and Medicine Dietary Reference Intakes (DRI) for Sodium (Na^+^) and Potassium (K^+^) Report concluded there remains insufficient evidence to establish a K^+^ DRI. This study tested the hypothesis that reduced Na^+^ and increased K^+^ excretion will positively associate with lower blood pressure in salt sensitive (SS) and salt resistant (SR) participants in the Dietary Approaches to Stop Hypertension Sodium Trial (DASH–Sodium). Via the NHLBI BioLINCC we accessed the DASH-Sodium dataset for data on systolic blood pressure (SBP), 24-h urinary Na^+^ and K^+^ excretion at screening (regular patient diet; *N* = 186, SS *N* = 222 SR) and post DASH diet (*N* = 71 SS, *N* = 119 SR). The relationships between SBP, urinary Na^+^ and K^+^ excretion, and Na^+^/K^+^ ratio were assessed via linear regression. At screening elevated urinary Na^+^ excretion positively associated with SBP in SS (1 g increase in urinary Na^+^ excretion = +1 0 ± 0.4 mmHg) but not SR participants, and urinary K^+^ excretion of <1 g K^+^/day was associated with higher SBP in SS and SR participants. Urinary K^+^ excretion ≥1 g/day, or a decreases in urinary Na^+^/K^+^ ratio, was not associated with lower SBP. Post the DASH–sodium diet intervention, SBP was reduced in SS and SR participants. However, no correlation was observed between reduced SBP and urinary K^+^ excretion or the urinary Na^+^/K^+^ ratio irrespective of the salt sensitivity of blood pressure. Our data support the DRI recommendation not to establish a K^+^ DRI and suggest further evidence is required to support a reduced Na^+^/K^+^ ratio to lower SBP.

## Introduction

Hypertension, the most common non-communicable disease worldwide, represents a significant global public health issue. Based on the 2017 American Heart Association (AHA) guidelines, the prevalence of hypertension among US adults is estimated to be 46% [1]; additionally, ~50% of hypertensive individuals are estimated to be salt sensitive (SS) [2]. As noted by the National Center for Chronic Disease Prevention and Health Promotion report [3] ~90% of American adults consume an excess of dietary sodium (Na^+^), with an average daily consumption exceeding 3400 mg in adult US males, a value almost three times the daily consumption recommended by the AHA [4] and the National Academy of Science, Engineering, and Medicine Dietary Reference Intakes (DRI) [5]. Given that excess dietary Na^+^ intake, which can drive the salt sensitivity of blood pressure and increase hypertension risk, global dietary Na^+^ intake is a public health risk. The impact of dietary Na^+^ intake on blood pressure has been investigated in multiple dietary intervention trials generating evidence that reduced dietary salt intake in controlled settings leads to reductions in blood pressure [6–8]. Further, meta-analyses have correlated dietary Na^+^ restriction with reductions in blood pressure suggesting there is a health benefit in both normotensive and hypertensive individuals irrespective of the salt sensitivity of blood pressure [9, 10].

Recent evidence suggests the salt sensitivity of blood pressure may be modulated, in part, by dietary potassium (K^+^) intake. Increasing dietary K^+^ intake appears to counteract the effects of dietary Na^+^ intake on increasing blood pressure [11–13]. Despite several studies that have documented blood pressure lowering effects of increasing K^+^ intake, the 2019 National Academy of Science, Engineering, and Medicine DRI for sodium and potassium Report did not establish a DRI for K^+^. This report concluded that more evidence is required to support a DRI of K^+^ with particular reference to a lack of K^+^ dose-response trials limiting the evidence to establish a K^+^ DRI [5]. Several studies have reported that the urinary Na^+^:K^+^ ratio has a stronger association with blood pressure than Na^+^ or K^+^ independently [14, 15]. Largely based on these data, a urinary Na^+^ to K^+^ molar ratio of <1 has been recommended [16, 17] as a beneficial target to improve long-term blood pressure control. Given the high global dietary Na^+^ intake this would necessitate dietary, or other means, of K^+^ supplementation—for which a DRI has not been established [5]. A leading dietary intervention study was the Dietary Approaches to Stop Hypertension 2 Trial (DASH-Sodium) conducted between 1997 and 2002 [18]. The DASH-Sodium trial was a multicenter, randomized clinical trial that examined the impact of three levels dietary Na^+^ intake in combination with either a control or DASH diet (rich in fruits, vegetables, and low-fat dairy products, and reduced in total fat) on blood pressure. This study demonstrated substantial effects of dietary Na^+^ reduction and the DASH diet on reducing blood pressure, with more significant blood pressure lowering effects with the combination of a DASH diet plus dietary Na^+^ reduction than dietary Na^+^ restriction alone in individuals with higher than optimal blood pressure [7]. Given that the DASH diet intervention elevated dietary K^+^ intake by increasing dietary intake of fruits and vegetables in combination with modifying daily dietary Na^+^ intake, examining the potential interaction between dietary Na^+^ and K^+^ intake on blood pressure in the DASH trial will provide valuable insight into the potential influence of dietary K^+^ on blood pressure.

The primary goal of this study was to analyze urinary Na^+^, K^+^ and the Na^+^:K^+^ excretion ratio, for associations with changes in systolic blood pressure (SBP) in participants from the DASH–Sodium trial during the initial screening period in which participants were consuming their regular diet without dietary intervention. The secondary goals of this study were to investigate: (a) the impact of the salt sensitivity of blood pressure on these responses and (b) the impact of the DASH–Sodium dietary intervention, which lowers SBP and increases dietary K^+^ intake, on these potential associations. Our analysis reports that in the DASH–Sodium study cohort: (1) a daily excretion of <1 g K^+^/day is associated with elevated SBP, (2) urinary K^+^ excretion of >1 g/day does not correlate with a reduction in SBP and, (3) a reduction in the urinary Na^+^:K^+^ excretion ratio is not associated with lower SBP irrespective of the salt sensitivity of blood pressure. Collectively our data support the recent DRI recommendation not to propose a DRI for K^+^ and suggest that further evidence is required to support the establishment of a Na^+^/K^+^ excretion ratio that would reduce SBP in the general population.

## Materials and methods

### Study design

**Fig. 1:**
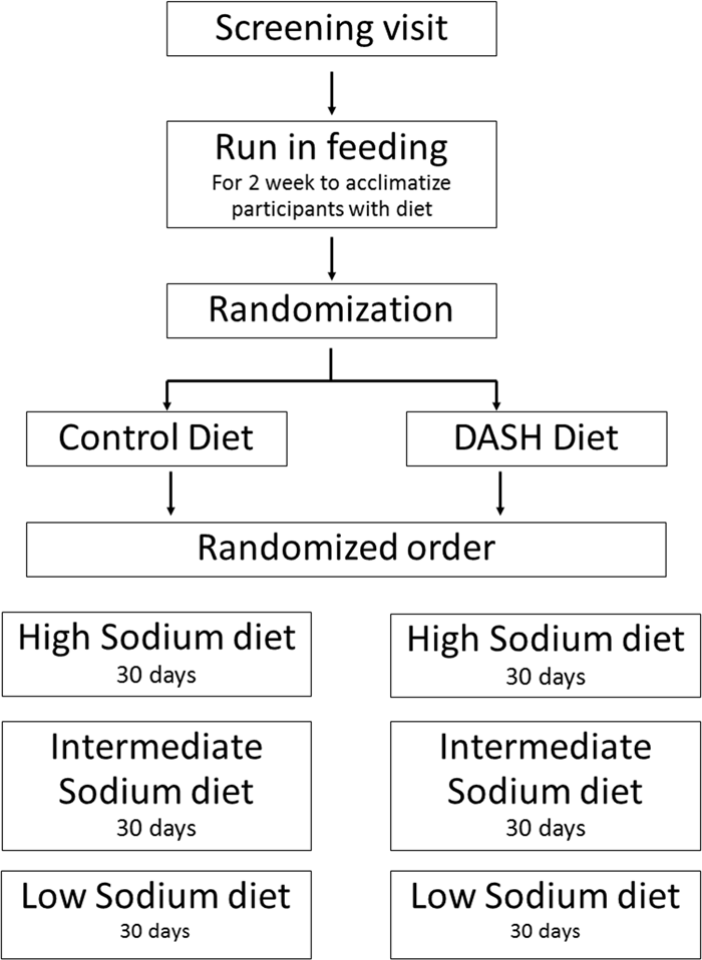
Study design of the Dietary Approaches to Stop Hypertension (DASH)–Sodium trial.

The National Heart, Lung and Blood Institute (NHLBI) Biologic Specimen and Data Repository Information Coordinating Center (BioLINCC) provided access to the DASH–Sodium trial data on urinary Na^+^, K^+^, and SBP values from study participants. The design of the DASH–Sodium trial has been described in detail previously [18]. In brief, and summarized in Fig. 1, a randomized control trial was conducted in 412 individuals who were healthy adults aged 22 years or older who were not taking any antihypertensive medications with a SBP of 120–159 mmHg and diastolic blood pressure (DBP) of 80–95 mmHg (range normal to Stage 1 hypertension). Following a screening phase and a 2-week run in period with a control diet, representing a typical American diet, study participants were randomized for the dietary intervention period to a control diet or a DASH diet that is rich in fruits, vegetables, low-fat dairy food and that increases K^+^ intake to ~120 mmol/day (4.7 g K^+^/day). Each dietary arm was further randomized in a crossover design resulting in each participant receiving their respective diet (control or DASH) containing low (LS; 50 mmol Na^+^/day—optimal daily Na^+^ intake), intermediate (IS; 100 mmol Na^+^/day—upper limit of daily Na^+^ intake recommendations), or high (HS; 150 mmol Na^+^/day—current average daily US Na^+^ intake) Na^+^ content, for 30 days each in a randomized order. Several measures, including incentives (e.g., cash and non-cash awards, personal encouragement), daily diary, and clinical staff monitoring ensured dietary compliance [18].

### Measurement of SBP and urinary sodium and potassium excretion

Twenty-four-hour ambulatory blood pressure recordings were taken during two screening periods and during the last nine days of each dietary intervention period. The data used for analysis represent the average cleaned SBP measured over each specified period. 24-h urine samples were obtained during screening and during the last week of each dietary intervention feeding period. Prior to storage at −80 °C urine samples were mixed to ensure a uniform sample and aliquoted in 5 ml volumes with 2 drops of 6 N HCl added per aliquot.

In the current study, we utilized screening values for SBP and urinary laboratory parameters from 222 SS and 186 of SR participants to evaluate Na^+^ and K^+^ interactions with baseline blood pressure. In order to assess potential interactions of urinary Na^+^ and K^+^ with SBP in response to changes from the HS to the LS DASH diet, we assessed SBP only, and urinary parameter values from participants from which all three urine samples (Screening, LS, and HS) were available for analysis. This resulted in the analysis of 71 SS and 119 SR participants.

### Data analysis

In the original DASH–Sodium trial, the baseline blood pressure used for analysis was the mean of value recorded during screening and the run-in period, and the blood pressure for the intervention period was the mean of the last five measurements at the end of each intervention. Baseline blood pressure and the clinical centers were represented as fixed effects, and the intervention periods were represented as random effects. The generalized estimating equation module of Stata was employed to compute power, and the residual variance estimate value and standard deviation for each estimate were provided. As has been previously reported the DASH–Sodium trial was sufficiently powered to detect changes evoked by the dietary interventions on SBP and DBP. The effects of Na^+^ reduction within the control diet and the DASH diet were assessed using the Holm method, and the resulting *P* value < 0.05 was determined to be significant [7]. All SBP and urinary Na^+^ and K^+^ excretion (mg/day) data obtained during the screening and run-in feeding period (referred to as baseline), and following 30-days LS and HS intake on either the control or DASH diet were provided to the authors via BioLINCC.

### Analytical variables

*Salt sensitive*: Participants with SBP being at least 5 mmHg higher after HS intake compared to the values after the LS intake; *Salt resistant*: Participants whose SBP differed by <5 mmHg between HS and LS intakes; *Independent variables*: Urinary Na^+^, Urinary K^+^ and Na^+^:K^+^ excretion ratio; *Dependent variable*: SBP; *Categorical variable*: Participants were categorized into groups on the basis of increments of 1 g/day in urinary Na^+^ and K^+^ excretion to evaluate the linear trends

### Statistical analysis

**Fig. 2:**
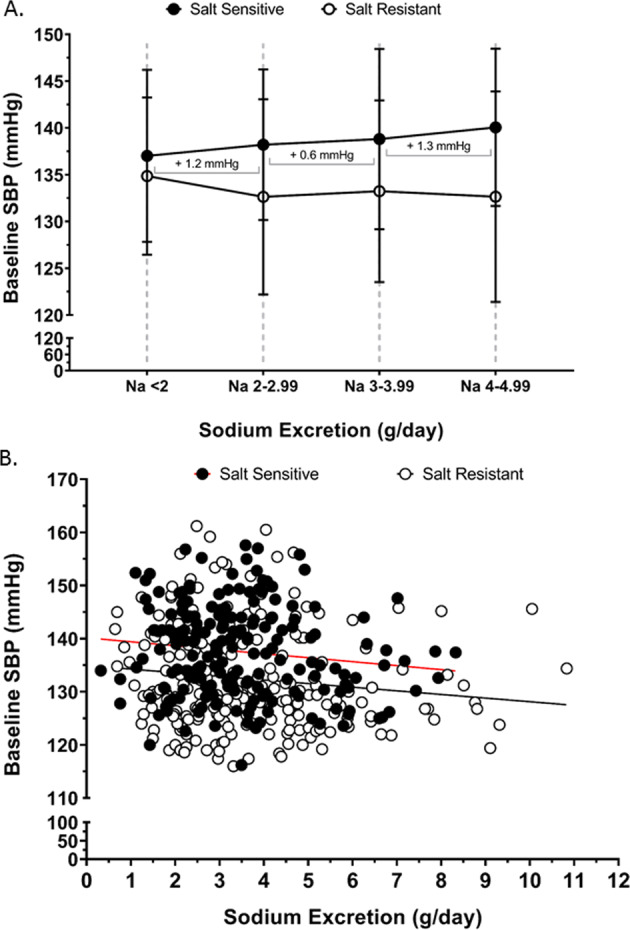
Association of urinary sodium excretion with systolic blood pressure. Baseline systolic blood pressure (SBP) at screening on regular diet (**a**) according to changes sodium excretion of <5 g/day, in salt sensitive (*n* = 154) and salt resistant (*n* = 180) group, slope of the for salt sensitive group is indicated for the sodium excretion ranges of <2–2.99 g/day, 3–3.99 g/day and 4–4.99 g/day (**b**) Correlation of baseline SBP (dependent variable) across the entire range of urinary sodium excretion (independent variable), (Pearson’s *R*^2^ for salt sensitive = 0.02 and for salt resistant = 0.02), in salt sensitive (*n* = 186) and salt resistant (*n* = 222) individuals.

**Fig. 3:**
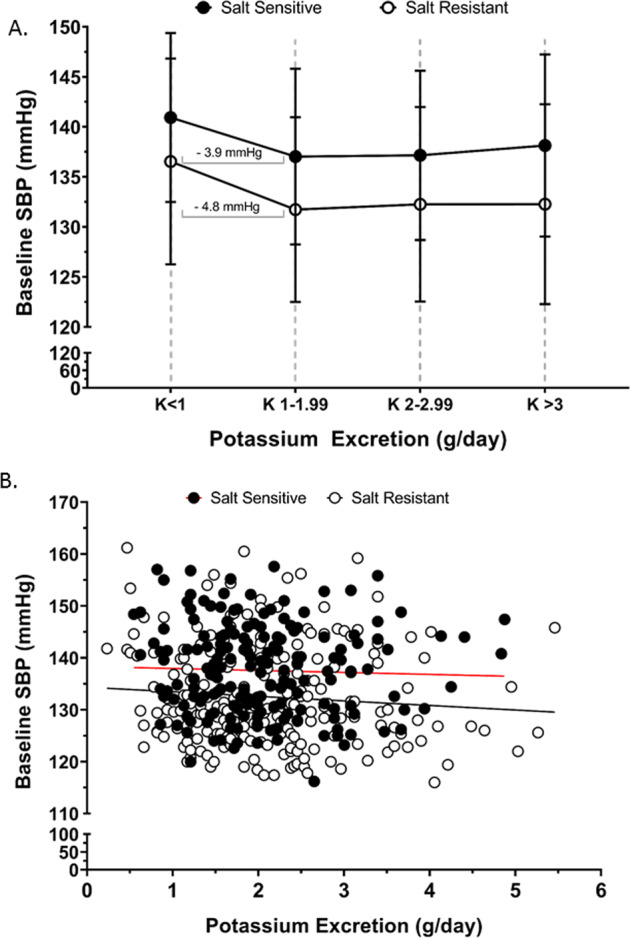
Association of urinary potassium excretion with systolic blood pressure. Baseline systolic blood pressure (SBP) at screening on regular diet (**a**) according to changes potassium excretion for salt sensitive (*n* = 186) and salt resistant (*n* = 222) group, slope for salt sensitive and salt resistant group is indicated for the potassium excretion range <1–1.99 g/day, values shown as mean ± SD. **b** Correlation of baseline SBP (dependent variable) across the entire range urinary potassium excretion (independent variable), (Pearson’s *R*^2^ for salt sensitive = 0.001 and salt resistant = 0.008), in salt sensitive (*n* = 186) and salt resistant (*n* = 222) individuals.

**Fig. 4:**
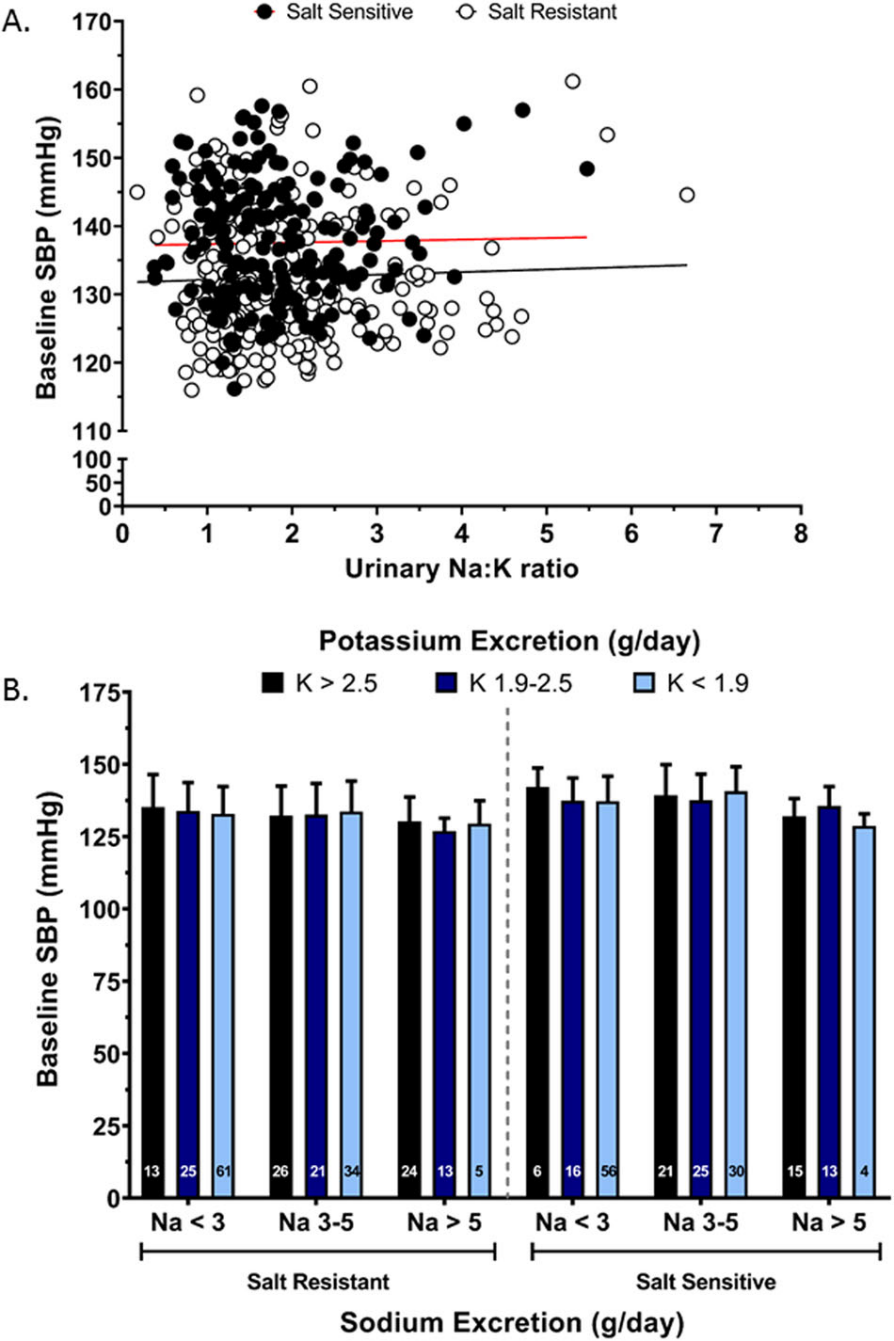
Association of urinary sodium to potassium excretion ratio with systolic blood pressure. Baseline SBP relative to urinary sodium to potassium excretion ratio (Na^+^/K^+^) (**a**) correlation of baseline SBP (dependent variable) across the entire range of urinary Na^+^/K^+^ excretion (independent variable), (Pearson’s *R*^2^ for salt sensitive = 0.0004 and salt resistant = 0.0016) (**b**) baseline SBP according to changes in sodium excretion and potassium excretion range in salt sensitive (*n* = 186) and salt resistant (*n* = 222) individuals at screening on their regular diet, values shown as mean ± SD. Data were analyzed by three-way ANOVA with pairwise comparison followed by Tukey’s post hoc test.

**Fig. 5:**
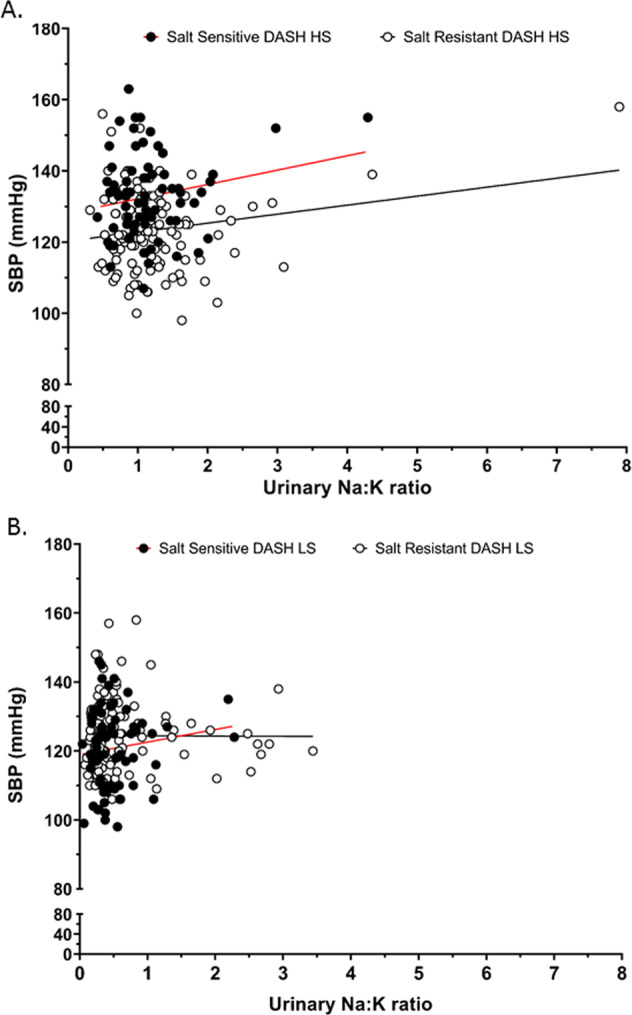
Association of urinary sodium to potassium excretion ratio with systolic blood pressure with dietary sodium intervention. Correlation of SBP values across the entire range of urinary sodium to potassium excretion (Na^+^/K^+^) ratio in salt sensitive (*n* = 71) and salt resistant (*n* = 119) individuals with dietary intervention of the Dietary Approaches to Stop Hypertension (DASH) (**a**) high sodium (HS), (Pearson’s *R*^2^ for salt sensitive = 0.04 and salt resistant = 0.04) (**b**) low sodium (LS) diet, (Pearson’s *R*^2^ for salt sensitive = 0.02 and salt resistant = 0.00002).

**Fig. 6:**
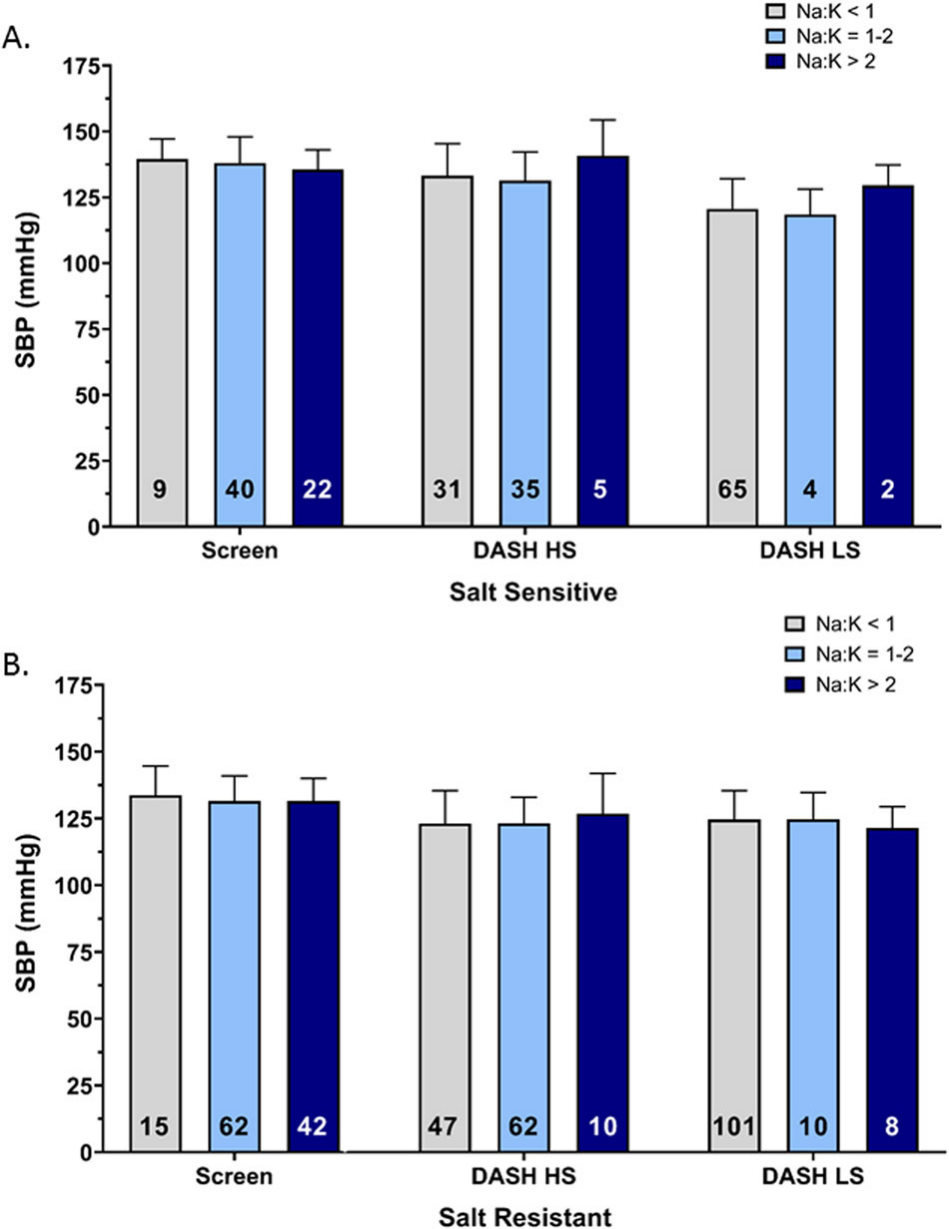
Comparative association of urinary sodium to potassium excretion ratio with systolic blood pressure with dietary sodium modification. Mean SBP relative to urinary sodium to potassium (Na^+^/K^+^) excretion ratio at the time of screening and with dietary intervention of Dietary Approaches to Stop Hypertension (DASH) high sodium (HS) and low sodium (LS) diet in (**a**) salt sensitive (*n* = 71), (**b**) salt resistant (*n* = 119) individuals, values shown as mean ± SD. Data were analyzed by three-way ANOVA with pairwise comparison followed by Tukey’s post hoc test.

**Fig. 7:**
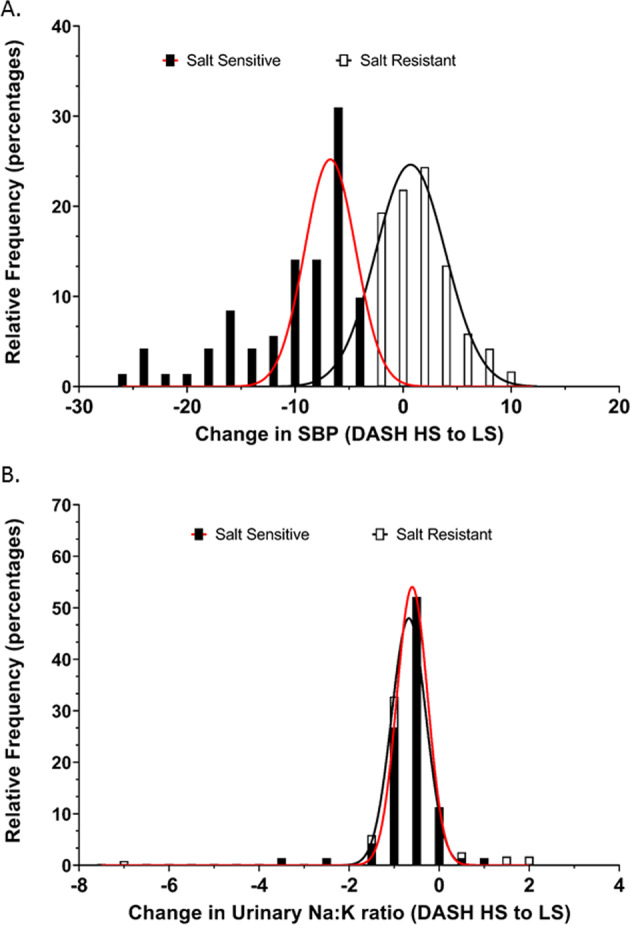
Frequency distribution of systolic blood pressure and urinary sodium to potassium excretion ratio with dietary sodium modification. Relative frequency distribution of (**a**) SBP changes, (Gaussian fit R^2^ for salt sensitive = 0.74 and salt resistant = 0.97) and (**b**) urinary Na^+^/K^+^, (Gaussian fit *R*^2^ for salt sensitive = 0.99 and salt resistant individuals = 0.99) in the group of salt sensitive (*n* = 71) and salt resistant (*n* = 119) individuals with changes from dietary intervention of Dietary Approaches to Stop Hypertension (DASH) high sodium (HS) diet to DASH low sodium (LS) diet.

Generalized linear models were employed for linear regression analysis (Pearson’s R correlation) to assess the association of urinary Na^+^ and K^+^ excretion with SBP. For Figs. 2 and 3, two-way ANOVA with Tukey’s post hoc was performed to compare SBP among the groups defined on the basis of urinary Na^+^ and K^+^ excretion. In order to quantify the effectiveness of increasing urinary Na^+^ and K^+^ excretion relative to SBP, Cohn’s Effect Size was calculated. In Figs. 4 and 5 generalized Pearson’s R correlation was performed to assess the association of the urinary Na^+^:K^+^ excretion ratio with SBP. In Fig. 6 data were analyzed by a three-way ANOVA and the pairwise comparisons were made using Tukey’s post hoc test. In Fig. 7 relative frequency distribution was assessed using a Gaussian fit analysis. In all analyses statistical significance was set at *p* < 0.05 (GraphPad prism software, version 8). Data are presented as mean ± SD.

## Results

### Participant demographics

Among study participants analyzed, 53% of SR and 62% of the SS participants were female, 51% of SR and 63% of SS participants were African-American (Table 1). The majority of participants were aged 31–55 years, college-educated, and employed fulltime. There were no significant differences in baseline characteristics for study participants across ethnicity or sex in either the SS or SR groups (Table 1).

**Table 1:** Baseline demographics across ethnicity and sex in salt sensitive and salt resistant individuals at the time of screening on their regular diet, values as percentage (*n*) for categorical variables and mean ± SDs for continuous variables.

	Salt resistant				Salt sensitive			
	African American		Non-African Americans		African American		Non-African Americans	
	Male	Female	Male	Female	Male	Female	Male	Female
Age % (*n*)								
18–30 y	2.5 (1)	1.3 (1)	6.3 (4)	4.5 (2)	0 (0)	2.2 (2)	2.4 (1)	0 (0)
31–55 y	85.0 (34)	87.8 (65)	73.4 (47)	77.3 (34)	75.0 (21)	75.5 (68)	54.8 (23)	53.8 (14)
≥56 y	12.5 (5)	10.8 (8)	20.3 (13)	18.2 (8)	25.0 (7)	22.2 (20)	42.9 (18)	46.2 (12)
Income % (*n*)								
≤$29,999	27.5 (11)	36.5 (27)	18.8 (12)	40.9 (18)	21.4 (6)	46.7 (42)	23.8 (10)	23.1 (6)
$30,000–$59,999	37.5 (15)	44.6 (33)	23.4 (15)	29.5 (13)	50.0 (14)	38.9 (35)	28.6 (12)	34.6 (9)
≥$60,000	35.0 (14)	16.2 (12)	57.8 (37)	25.0 (11)	28.6 (8)	12.2 (11)	42.9 (18)	34.6 (9)
Education % (*n*)								
High school	17.5 (7)	16.2 (12)	6.3 (4)	18.2 (8)	17.9 (5)	26.7 (24)	7.1 (3)	23.1 (6)
Some college	42.5 (17)	41.9 (31)	31.3 (20)	25.0 (11)	35.7 (10)	43.3 (39)	21.4 (9)	42.3 (11)
College graduate	27.5 (11)	27.0 (20)	25.0 (16)	25.0 (11)	21.4 (6)	21.1 (19)	23.8 (10)	7.7 (2)
Postgraduate/degree	12.5 (5)	14.9 (11)	37.5 (24)	29.6 (13)	21.4 (6)	8.9 (8)	47.6 (20)	26.9 (7)
Employment % (*n*)								
Full time	35.0 (14)	51.4 (38)	32.8 (21)	36.4 (16)	53.6 (15)	53.3 (48)	33.3 (14)	53.8 (14)
Part time	20.0 (8)	16.2 (12)	26.6 (17)	22.7 (10)	28.6 (8)	17.7 (16)	28.6 (12)	19.2 (5)
Retired	12.5 (5)	6.8 (5)	9.4 (6)	11.4 (5)	7.1 (2)	10.0 (9)	9.5 (4)	11.5 (3)
Other	30.0 (12)	25.7 (19)	29.7 (19)	25.0 (11)	10.7 (3)	17.7 (16)	26.2 (11)	11.5 (3)
Hypertension (SBP > 140) % (*n*)								
No	82.5 (33)	71.6 (53)	82.8 (53)	75.0 (33)	60.7 (17)	53.3 (48)	69.0 (29)	61.5 (16)
Yes	17.5 (7)	28.4 (21)	17.2 (11)	25.0 (11)	39.3 (11)	46.7 (42)	31.0 (13)	38.5 (10)
Weight (Kg)	90.4 ± 12.6	82.3 ± 12.8	90.1 ± 14.0	76.3 ± 16.5	90.4 ± 12.9	79.1 ± 15.5	91.6 ± 13.6	75.1 ± 15.1
Height (cm)	178.2 ± 6.3	163.3 ± 5.7	178.0 ± 4.9	163.0 ± 6.3	177.4 ± 7.0	163.8 ± 6.1	177.5 ± 6.7	163.2 ± 4.4
BMI (Kg/m^2^)	28.5 ± 3.7	30.9 ± 5.0	28.4 ± 4.2	28.6 ± 5.5	28.8 ± 3.9	29.4 ± 5.2	29.0 ± 3.8	28.2 ± 5.6
Waist circumference (cm)	99.4 ± 10.9	97.7 ± 13.2	101.5 ± 11.3	92.5 ± 13.9	100.6 ± 10.9	94.6 ± 14.2	104.6 ± 12.3	95.0 ± 13.9

Demographic information regarding income, education, and employment were missing for a few of the participants. Data were analyzed using two-way ANOVA with pairwise comparison and Tukey post hoc.

### Association of baseline daily sodium and potassium excretion with SBP

Baseline SBP, assessed during the screening visit prior to dietary intervention was significantly higher in SS (137.6 ± 8.7 mmHg) vs. SR participants (132.5 ± 9.6 mmHg; *p* < 0.05, Table 2). In contrast there was no significant difference in 24 h urinary Na^+^ excretion, 24 h urinary K^+^ excretion and the urinary Na^+^:K^+^ ratio between SS and SR participants at screening (Table 2). Further, there was no significant effect of sex or  ethnicity on these variables, as such subsequent analyses were not adjusted for age or  ethnicity. In SS, but not SR participants, each additional g/day in urinary Na^+^ excretion across the range of <2 g/day to 5 g/day resulted in a higher SBP value of approximately 1.0 ± 0.4 mmHg in SBP/g Na^+^ excretion (Fig. 2a). The measures >5 g/day Na+ were not included due to increased sample variability. When assessed by linear regression across the entire range of observed Na^+^ excretion we observed no correlation between urinary Na^+^ excretion and SBP in either SS (*R*^2^ = 0.02) or SR (*R*^2^ = 0.02) participants (Fig. 2b). In both SS and SR participants urinary K^+^ excretion of <1 g/day elevated SBP by 3.9 and 4.8 mmHg respectively vs. SBP values obtained for urinary excretion of 1–1.99gK^+^/day (Fig. 3a) and the Cohen’s D score for the difference in the SBP among the participants with less than 1 g/day versus 1-1.9 g/day of urinary K^+^ excretion showed a medium effect size in both SS (0.45) and the SR (0.49) group. However, when assessed across the entire range of observed K^+^ excretion we observed no correlation between K^+^ excretion and SBP in either SS (*R*^2^ = 0.001) or SR (*R*^2^ = 0.008) participants (Fig. 3b). Further, we observed no association between the urinary Na^+^:K^+^ ratio and SBP and no impact of urinary K^+^ excretion across any dietary Na^+^ excretion range on SBP in either SS (*R*^2^ = 0.004) or SR (*R*^2^ = 0.002) participants (Fig. 4a, b).

**Table 2:** Baseline parameters across ethnicity and sex in salt sensitive and salt resistant individuals at the time of screening on their regular diet, values shown as mean ± SD.

	Salt resistant				Salt sensitive			
	African American		Non-African Americans		African American		Non-African Americans	
	Male	Female	Male	Female	Male	Female	Male	Female
*N*	40	74	64	44	28	90	42	26
SBP (mmHg)	129.6 ± 9.3	134.8 ± 9.2	131.1 ± 9.0	133.2 ± 10.8	138.6 ± 9.9*	137.8 ± 9.0*	135.9 ± 6.6*	138.1 ± 9.4*
Urinary Na:K (g/day)	2.6 ± 1.2	2.0 ± 1.1	1.6 ± 0.7	1.7 ± 0.9	2.0 ± 0.7	1.7 ± 0.8	2.0 ± 0.7	1.9 ± 1.0
Urinary Na (g/day)	4.6 ± 2.2	3.1 ± 1.8	4.1 ± 1.8	3.0 ± 1.2	4.2 ± 1.9	3.0 ± 1.2	4.0 ± 1.5	3.7 ± 1.8
Urinary K (g/day)	2.0 ± 1.0	1.7 ± 0.8	2.7 ± 1.0	2.0 ± 0.8	2.1 ± 0.8	2.0 ± 0.8	2.2 ± 1.0	2.1 ± 0.8
Urinary creatinine (g/day)	2.1 ± 0.1	1.4 ± 0.1	1.7 ± 0.1	1.1 ± 0.0	2.0 ± 0.1	1.4 ± 0.0	1.7 ± 0.1	1.0 ± 0.1

Data were analyzed using two-way ANOVA with pairwise comparison and Tukey post hoc.

*p < 0.05 vs. salt resistant of similar ethnicity and sex.

### Impact of DASH diet on the association of urinary sodium to potassium excretion ratio with SBP

Within the sub group of SS participants randomly assigned to DASH-Sodium dietary intervention arm (*N* = 71) there was a significant (*p* < 0.05) reduction in SBP on the DASH-LS diet compared to the baseline screening SBP value (Table 3). In the sub group of SR participants randomly assigned to the DASH-Sodium intervention (*N* = 119) there were significant (*p* < 0.05) reductions in SBP on both the DASH-HS and DASH-LS diets compared to the baseline screening SBP value (Table 3). On the DASH-Sodium diet, following both the LS and HS interventions compared to screening there was a significant (*p* < 0.05) increase in urinary K^+^ excretion and reduction in the urinary Na^+^:K^+^ ratio (that was greater during the LS intervention), in both SS and SR participants (Table 3).

**Table 3:** Parameters in the same set of salt sensitive and salt resistant individuals across the change in their diet at the time of screening on their regular diet (SCREEN), with dietary intervention of Dietary Approaches to Stop Hypertension (DASH) high sodium (HS) and low sodium (LS) diet, values shown as mean ± SD.

	Salt resistant			Salt sensitive		
	SCREEN	DASH HS	DASH LS	SCREEN	DASH HS	DASH LS
*N*	119	119	119	71	71	71
SBP (mmHg)	131.8 ± 9.2	123.5 ± 11.2^#^	124.4 ± 10.5^#^	137.5 ± 9.0*	132.8 ± 11.7*	120.7 ± 11.3*^#^
Urinary Na:K (g/day)	1.9 ± 0.9	1.3 ± 0.8^#^	0.6 ± 0.7^#^	1.8 ± 0.8	1.2 ± 0.6^#^	0.5 ± 0.4^#^
Urinary Na (g/day)	3.7 ± 1.9	3.4 ± 1.3	1.6 ± 1.2^#^	3.5 ± 1.6	3.2 ± 1.3	1.4 ± 0.8^#^
Urinary K (g/day)	2.2 ± 0.9	3.0 ± 1.0^#^	3.2 ± 1.1^#^	2.1 ± 0.9	3.0 ± 1.1^#^	3.2 ± 1.2^#^

Data were analyzed using two-way ANOVA with pairwise comparison and Tukey post hoc.

*p < 0.05 vs. salt resistant group with similar dietary intervention.

^#^*p* < 0.05 vs. screening within the salt sensitive and salt resistant group.

Significantly, we observed no association between the urinary Na^+^:K^+^ ratio and SBP on the DASH HS or DASH LS dietary intervention in either SS (DASH HS *R*^2^ = 0.04, DASH LS *R*^2^ = 0.02) or SR (DASH HS *R*^2^ = 0.04, DASH LS *R*^2^ = 0.00002) participants (Fig. 5a, b). The DASH dietary intervention significantly increased the number of participants in both SS and SR groups with a urinary Na^+^:K^+^ ratio of <1 on both the HS and LS diet. However, the urinary Na^+^:K^+^ had no impact on SBP within dietary intake groups (Fig. 6a, b). Further, when expressed as a frequency distribution histogram the change in SBP from the DASH HS to LS dietary intervention exhibits a profound leftward shift in the SS group compared to SR group (Fig. 7a). In contrast, the frequency distribution histogram for change in the urinary Na^+^:K^+^ ratio from the DASH HS to LS dietary intervention shows no difference in the Gaussian curve and distribution between SS and SR participants (Fig. 7b).

## Discussion

In the current study, using data from the DASH–Sodium trial, during screening when participants are consuming their normal dietary intake, we report a slope increment of an elevation in SBP of approximately 3 mmHg across the urinary Na^+^ excretion range of 2–5 g/day in SS, but not SR participants. However, when assessed across the full range of observed urinary Na^+^ excretion values we did not observe a positive correlation between SBP and urinary Na^+^ excretion in either SS or SR participants. Significantly, despite urinary K^+^ excretion of <1 g K^+^/day associating with higher SBP in SS and SR participants further increments in urinary K^+^ excretion did not correlate with a reduction in SBP in either participant group. Furthermore, at baseline screening we did not observe a correlation between the urinary Na^+^:K^+^ excretion ratio irrespective of the salt sensitivity of blood pressure. Following the DASH dietary intervention we observed no correlation between a urinary Na^+^:K^+^ ratio and SBP in either SS or SR participants. As such our data, from the DASH–Sodium Trial, in US participants at both baseline screening and following a highly controlled dietary intervention does not support the hypothesis that a reduced urinary Na^+^:K^+^ ratio will be beneficial in population level blood pressure reduction or support the proposal for a urinary Na^+^:K^+^ molar ratio of <1 to lower blood pressure.

In contrast to the PURE [19], INTERSALT [20], and INTERMAP [21] studies, that established a population level positive association between urinary Na^+^ excretion and blood pressure, the DASH–Sodium Trial enables the establishment of the salt sensitivity of blood pressure in trial participants. In SR participants we observed no relationship between urinary Na^+^ excretion and SBP. In contrast, in SS participants we observed a slope increment of an increase in SBP of 1.3 mmHg for each 1 g increase in urinary Na^+^ excretion across the excretion range of 3–5 g Na^+^/day which is within typical average range of daily Na^+^ intake in the US [22]. In contrast, when assessed across the whole range of observed urinary Na^+^ excretion, we observed no association between urinary Na^+^ excretion and SBP in either SS or SR participants. We speculate this discrepancy between a positive relationship between SBP and urinary Na^+^ excretion within the expected range of dietary Na^+^ excretion of 3–5 g/day and no association over the complete range of values reflects the impact of multiple participants in the DASH–Sodium study exhibiting high levels of urinary Na^+^ excretion, greater than 5 g/day, and comparatively low blood pressure. Significantly, the value obtained in this study for an increase in SBP within 3–5 g/day Na^+^ excretion is comparable to that obtained in the PURE study which reported a positive slope increment of a 1.7 mmHg increase in SBP per 1 g increase in urinary Na^+^ excretion across the same range of Na^+^ excretion values [23]. The difference between the observed increase in SBP in response to elevated urinary Na^+^ excretion between DASH-Sodium and PURE may reflect (1) significant differences in sample size and racial backgrounds of the participants and (2) the potential differences in methods to assess urine content of 24-h urine collection compared to an estimation from a single morning spot urine sample in the DASH-Sodium versus PURE Study respectively. Our data support guidelines to limit dietary Na^+^ intake [5, 24] and suggest that reduced dietary salt intake may only lower SBP in SS patients.

The influence of K^+^ intake on blood pressure remains controversial, with conflicting data emerging from multiple clinical studies [25]. In a randomized controlled trial conducted in free living non-dietary regulated participants with a mean SBP of 132 mmHg and not taking blood pressure lowering medication, K^+^ intake was increased by dietary intake (via fruit and vegetable intake) or direct K^+^ supplements. In this study increased K^+^ intake up to 40 mmol/day had no impact on blood pressure [22, 26]. A separate randomized placebo-controlled crossover trial was conducted in participants who have never received antihypertensive medication with mildly elevated blood pressure [27]. Participants were maintained on their normal diet and received K^+^ at 64 mmol/day for a 4-week period as either potassium chloride or bicarbonate—in this study there was no effect of K^+^ supplementation on office blood pressure [27]. In contrast in a randomized placebo-controlled, crossover study, in which untreated patients with a mean SBP of 145 mmHg blood pressure received 4 weeks of supplemental K^+^ at 3 g/day and a diet relatively low in Na^+^ reported a reduction in SBP of 3.9 mmHg. Beyond the highly controlled trials discussed above the PURE study reports that for each increment of 1 g/day of urinary K^+^ excretion there is a reduction of 0.75 mmHg in SBP across the excretion range of <1.25 to 3 g K^+^/day [23]. In the DASH–Sodium data, we observed an elevation in SBP in both SS and SR participants when urinary K^+^ excretion was below 1 g/day. However, we did not observe any correlation between urinary K^+^ excretion and SBP or an impact of urinary K^+^ excretion on SBP over the range of <1 to >3 g K^+^ excretion per day. We speculate that discrepancy between the PURE study data and our own analysis of the DASH-Sodium data may reflect the difference in SBP response to urinary K^+^ excretion reported in PURE between Chinese and non-Chinese participants. Chinese participants exhibited a large reduction in SBP with increased urinary K^+^ excretion versus a smaller SBP effect in participants from the rest of the world. As the DASH-Sodium trial did not contain Chinese participants this may have influenced the outcome.

Further, several studies have suggested that the blood pressure reduction evoked by K^+^ intake may be dependent on dietary Na^+^ intake [28, 29]. In our analysis of the DASH-Sodium dataset we observed no association with urinary K^+^ excretion and SBP, during the patient screening visit or during DASH dietary intervention when Na^+^ intake was modified, suggesting an independence of the effects of Na^+^ and K^+^ on SBP in this study. The 2019 DRI Report concluded that there is insufficient evidence on the effects of K^+^ on blood pressure and did not establish a DRI of K^+^ [5]. Our data support the 2019 DRI Report and suggests that dietary K^+^ supplementation may not significantly reduce blood pressure in the general population.

At present there is conflicting evidence regarding the potential blood pressure lowering effects of a reduction in urinary Na^+^/K^+^ excretion. The TAIM randomized control trial in hypertensive participants on a pharmacological intervention reported no significant difference in DBP between the control diet group and a low Na^+^/high K^+^ diet group during a six month period [30]. This suggests long-term reductions in the urinary ratio do not lower blood pressure. Despite several prior studies reporting an association between blood pressure and the urinary Na^+^/K^+^ ratio there are several limitations to be considered. A multicenter study by Suppa et al. conducted in hypertensive participants reported a significant reduction in SBP following 4 weeks of modified low sodium high potassium salt intake compared to participants receiving a normal salt intake [31], yet all participants were receiving a beta blocker, which is not standard first line treatment for hypertension [1]. Further, the INTERSALT study, which documented a positive association between the urinary Na^+^/K^+^ ratio and blood pressure reported a loss of statistical significance of the correlation in 44 of the 52 centers after adjustment for covariates including age, sex and BMI [32, 33]. In addition, a cross-sectional study, the Dallas heart study, which reported an increase in SBP for an increase in the urinary Na^+^/K^+^ ratio is limited by the modest correlation the use of single morning urine sample [34]. The PURE study reported a strong linear association between estimated Na^+^/K^+^ ratio and SBP, that was maintained after covariate adjustment, with the greatest SBP observed with the highest estimated K^+^ and lowest estimated K^+^ excretion [23]. Although we observed that a daily K^+^ excretion of <1 g/day is associated with elevated SBP we observed no association between the urinary Na^+^/K^+^ excretion ratio and SBP at screening or following DASH-dietary intervention in SS or SR participants. In conjunction with the study by Zanetti et al. [35], our data suggest high Na^+^ and low K^+^ excretion may increase the likelihood of having increased SBP. However, the lack of association between urinary Na^+^/K^+^ ratio and SBP in our data do not support a urinary Na^+^/K^+^ molar excretion ratio of 1:1 as a mechanism to lower blood pressure [16, 17].

The current study has several strengths: (1) The DASH -Sodium trial was a carefully controlled feeding study and compliance was continuously monitored, (2) The crossover design for Na^+^ intervention allowed participant’s to serve as their own control and diminished inter-person variability, (3) 24-h ambulatory blood pressure recordings were taken, (4) absence of the confounding effects of antihypertensive mediations, and (5) 24-h urine sample collection. The major limitation of the present study is the relatively modest sample size that may have decreased our statistical power to detect modest effects of urinary Na^+^, K^+^, and Na^+^:K^+^ ratio on SBP. While we did not observe an imbalance in baseline characteristics, residual confounding in our analysis is possible.

In conclusion the current analysis of the DASH–Sodium Trial demonstrates that dietary K^+^ supplementation is not associated with lower SBP and that the reductions in SBP observed following DASH dietary intervention occurred independently of a decrease in the urinary Na^+^/K^+^ excretion ratio irrespective of the salt sensitivity of blood pressure. Our data do not support the establishment of a 1:1 molar excretion ratio of Na^+^/K^+^ as blood pressure lowering strategy in US African American and non-African Americans and support the DRI recommendation not to propose daily K^+^ intake guidelines. Given the limitations of the current analysis and the ongoing controversy regarding the role of dietary K^+^ on blood pressure future carefully controlled studies are required to elucidate the potential impact of dietary K^+^ and urinary Na^+^/K^+^ excretion ratio on blood pressure in both hypertensive and normotensive SS and SR participants.

### Summary

#### What is known about the topic

- Several clinical trials, including the Dietary Approaches to Stop Hypertension (DASH) – Sodium trial have established significant effects of dietary sodium reduction on lowering blood pressure.
- Increased potassium intake in combination with reduced sodium intake appears to lower blood pressure and a urinary sodium to potassium molar ratio of <1 has been proposed to improve blood pressure control.
- Although potassium potentially modulates blood pressure, the 2019 National Academy of Science, Engineering, and Medicine Dietary Reference Intakes for Sodium and Potassium Report states that more evidence is required to establish a daily potassium intake level.

#### What this study adds

- Urinary potassium excretion ≥1 g/day does not correlate with lower SBP irrespective of the salt sensitivity of blood pressure in DASH–Sodium trial participants supporting the recent DRI recommendations not to propose potassium intake guidelines.
- The reduction in SBP in salt sensitive and salt resistant individuals following the DASH dietary intervention occurred independently of a reduction in urinary sodium to potassium excretion ratio. These findings do not support the establishment of 1:1 molar excretion ratio of urinary sodium to potassium for blood pressure reduction.
